# Retraction: *In vivo* experiments demonstrate the potent antileishmanial efficacy of repurposed suramin in visceral leishmaniasis

**DOI:** 10.1371/journal.pntd.0013690

**Published:** 2025-11-11

**Authors:** The PLOS Neglected Tropical Diseases Editors

After this article [1] was published, concerns were raised regarding results presented in Figs 2 and 5.

Specifically:

- In Fig 2:
  - Multiple areas between the FACS panels appear more similar than would be expected from independent results
  - There appear to be repetitive areas within the Control and Suramin 200 µM panels
- In Fig 5, the DNA bands between 400 and 100 bp appear similar across lanes 2–4

Corresponding author RB disagreed with the above concerns. They provided some of the raw FACS data files, details of the gating strategy underlying Fig 2, and replicate data from the time of the original experiments for Fig 5. PLOS does not consider the above concerns to be resolved in the absence of the full original underlying data for the figures of concern. Additionally, editorial assessment of the provided FACS data raised further concerns for the reliability and validity of the published results.

In light of the above concerns, the *PLOS Neglected Tropical Diseases* Editors retract this article.

SK, SKJ, JJJ, SG, and RB did not agree with the retraction and stand by the article’s findings. DD did not agree with the retraction. JD responded but expressed neither agreement nor disagreement with the editorial decision. SD, SAN, and SM either did not respond directly or could not be reached.
